# Vitamin D_3_ alleviates intestinal injury in necrotizing enterocolitis and lipopolysaccharide-induced inflammatory response in dendritic cells in rats

**DOI:** 10.55730/1300-0144.5895

**Published:** 2024-04-15

**Authors:** Bo KE, Chaoyu LI, Song LI, Jingjing YAN, Leke SUN

**Affiliations:** 1 Department of Hematology, Jiangxi Provincial People’s Hospital, The First Affiliated Hospital of Nanchang Medical College, Nanchang, China; 2 The Jiangxi Province Key Laboratory of Hematologic Diseases, Nanchang, China; 3 Department of Pediatrics, Jiangxi Provincial People’s Hospital, The First Affiliated Hospital of Nanchang Medical College, Nanchang, China; 4 Jiangxi Medical College Nanchang University, China; 5 Department of Pediatrics, Nanchang People’s Hospital, Nanchang, China

**Keywords:** Necrotizing enterocolitis, vitamin D_3_, dendritic cells, inflammatory response

## Abstract

**Background/aim:**

Necrotizing enterocolitis (NEC) is a serious condition that predominantly affects premature infants and involves an aberrant immune response and inflammatory cytokine release resulting in intestinal epithelial damage. The current study investigated the immunoregulatory effects of vitamin D_3_ on the maturation and activation of dendritic cells (DCs) and the antiinflammatory impact on the intestines in a neonatal rat model of NEC.

Materials and methods: Inflammatory damage to intestinal tissue was assessed via morphological changes and apoptosis and DC expression of costimulatory molecules, inflammatory factors, and immunoregulatory factors by immunohistochemical staining, quantitative real-time PCR, and immunofluorescence. The fluorescein isothiocyanate-ovalbumin (FITC-OVA) uptake assay was used to analyze DC endocytosis.

**Results:**

Vitamin D_3_ administration attenuated intestinal damage and apoptosis, inhibiting CD86 and increasing CD80 expression. Lipopolysaccharide (LPS)-challenged DC2.4 cells in vitro showed upregulated CD86, tumor necrosis factor*-*α (TNF-α), interleukin*-*1β (IL-1β), inducible nitric oxide synthase (iNOS), and indoleamine 2,3-dioxygenase 1 (IDO-1) expression, which were all reduced by vitamin D_3_, except for IDO-1. LPS inhibited CD80 expression, which was restored by vitamin D_3_ treatment, and endocytic capacity was improved. Vitamin D_3_ ameliorated intestinal damage in neonatal rats with NEC and exerted antiinflammatory and immunomodulatory effects on DCs.

**Conclusion:**

Vitamin D_3_ has potential as a supplementary treatment for NEC patients.

## 1. Introduction

Necrotizing enterocolitis (NEC) is an inflammatory intestinal disease with high morbidity and mortality, caused by the failure of premature neonates to adapt to enteral nutrition. NEC occurs in approximately 5% of infants admitted to neonatal intensive care units, with an incidence of 9% among infants born at a gestational age of 22–29 weeks [1]. The vast majority (>90%) of NEC cases occur in preterm infants, especially those who are of very low birthweight of <1500 g. The incidence of NEC is inversely proportional to gestational age at birth, and the time to onset of NEC is longer in more premature infants [2]. The mortality rate of NEC among extremely premature infants was reported as 30% [3]. NEC has multifactorial causes and is associated with various prenatal and postnatal factors [4]. The disorder is characterized by abdominal distension, vomiting, bloody stool, and sepsis with intestinal wall and portal vein pneumatosis visible on imaging [5,6].

The etiopathogenesis of NEC remains unclear but an immature intestinal barrier, altered microbiota, intestinal immaturity [7], enteral feeding with formula milk [8], bacterial colonization, hypoxic-ischemic damage, and aberrant toll-like receptor 4 (TLR4)-mediated intestinal inflammatory response are contributing factors [9]. The excessive inflammatory response may lead to intestinal mucosal edema, damage, and intestinal wall necrosis [10]. Treatment involves intestinal rest, abdominal decompression, and broad-spectrum antibiotics, but peritoneal drainage, bowel resection, and enterostomy may be necessary in severe cases [11]. The therapeutic value of probiotics [12], stem cell transplantation [13,14], exosomes, and targeted treatment of intestinal ischemia with heparin-binding epidermal growth factor-like growth factor have all been studied [15,16].

Vitamin D_3_ is a secosteroid hormone with roles in calcium homeostasis and bone growth that promotes collagen matrix mineralization [17]. It is absorbed from the diet or synthesized from 7-dehydrocholesterol upon exposure of the skin to sunlight. Inactive vitamin D_3_ is hydroxylated by hepatic cytochrome P450 (CYP2R1), generating 25-hydroxyvitamin D (25(OH)D), which is converted into 1,25-dihydroxy vitamin D_3_ (1,25(OH)_2_D_3_) by the hydroxylation of CYP27B1 [18,^19^]. Active vitamin D_3_ binds the vitamin D receptor (VDR), consisting of ligand-binding and DNA-binding domains, and causes transcriptional activation or repression mediated by the 1,25(OH)_2_D_3_-VDR complex [20]. The regulatory roles of vitamin D in cellular proliferation, differentiation, apoptosis, angiogenesis, and immunological responses have been reported and deficiency is associated with many diseases, including multiple sclerosis [21], type I diabetes [22], asthma [23], and autoimmune diseases [24]. The involvement of vitamin D in immunoregulation, epithelial cell function, and metabolic regulation has been scrutinized, but immune regulatory effects in neonatal rats with NEC remain uninvestigated. The impacts of vitamin D_3_ on intestinal damage in a rat model of neonatal NEC and on dendritic cell (DC) maturation and activation were investigated in the present study.

## 2. Materials and methods

### 2.1. Animals

Thirty neonatal Sprague-Dawley rats were obtained from Jiangxi University of Chinese Medicine (Nanchang, China) and randomly divided into control, NEC, and vitamin D_3_-treated groups. Animals were fed 0.1 mL of animal formula milk every 4 h via an orogastric tube adapted from a peripherally inserted central catheter. NEC and vitamin D_3_-treated animals were subjected to hypoxia (100% nitrogen for 1 min) followed by hypothermia (4 °C for 10 min) twice a day beginning immediately after birth until the end of the experiment [25]. Vitamin D_3_-treated rats were given daily vitamin D_3_ at 2000 IU/kg body weight. At a dose of about 2000 IU/day, the calcium and phosphorus levels of the animals did not change during the follow-up period [26]. Animals were euthanized after 72 h. Ethical approval was granted by the Ethics Committee of Jiangxi Provincial People’s Hospital (Approval No. KT051).

### 2.2. Reagents and antibodies

Esbilac animal formula milk was purchased from PetAg (Hampshire, IL, USA). Vitamin D drops were purchased from Sinopharm Xingsha Pharmaceuticals (Xiamen, China). H&E staining regent (G1001), a TUNEL cell apoptosis detection kit (G1501), DAB (G1212), phosphate-buffered saline (PBS; G4202), and RPMI-1640 medium (G4531) were purchased from Servicebio (Wuhan, China). TRIeasy Total RNA Extraction Reagent (10606ES60), Hifair III 1st Strand cDNA Synthesis SuperMix (11139ES60), and Hieff qPCR SYBR Green Master Mix (11201ES08) were purchased from Yeasen (Shanghai, China). Vitamin D_3_ (V8070), ovalbumin (OVA)-FITC (SF069), and DAPI (C0065) were purchased from Solarbio Life Sciences (Beijing, China). DC2.4 mouse DCs were purchased from Fenghui Biotechnology (Changsha, China). Fetal bovine serum (FSP500) was purchased from ExCell Bio (Shanghai, China). LPS (*Escherichia coli* O55:B5) was purchased from Sigma-Aldrich (St. Louis, MO, USA). Brefeldin A (M2294) was purchased from AbMole Bioscience (Houston, TX, USA). CD80 antibody, CD86 antibody, tumor necrosis factor-α (TNF-α) antibody, interleukin-1β (IL-1β) antibody, inducible nitric oxide synthase (iNOS) antibody, and indoleamine 2,3-dioxygenase 1 (IDO-1) antibody were purchased from Proteintech (Wuhan, China). Horseradish peroxidase (HRP)-conjugated anti-IgG antibody was purchased from MXB Biotechnologies (Fuzhou, China). Cy3-conjugated IgG secondary antibodies were purchased from ABclonal (Wuhan, China).

### 2.3. Cell culture

Mouse dendritic DC2.4 cells were grown in RPMI-1640 containing 10% fetal bovine serum at 37 °C in a 5% CO_2_ incubator (Thermo, Rochester, NY, USA). Vitamin D_3_ was dissolved in physiological saline and LPS in RPMI-1640 as a 100 mM stock solution and stored at −80 °C.

### 2.4. Hematoxylin and eosin (H&E) staining

Intestinal specimens were dissected and fixed with 10% formalin solution for 24 h, embedded in paraffin, and sliced into sections of 3–5 μm for H&E staining. Stained sections were examined by a blinded observer under an optical microscope and morphological changes were graded as normal, mild, or moderate according to Barlow’s protocol [27].

### 2.5. TUNEL assay

The FITC TUNEL cell apoptosis detection kit was used to measure rates of apoptosis. Briefly, paraffin-embedded samples were dewaxed and incubated with proteinase K at 37 °C for 30 min. An equilibration buffer was added to cover the sample and slides were incubated at room temperature for 10 min. TdT incubation buffer (recombinant TdT enzyme FITC-12-dUTP, labeling mix, and equilibration buffer = 1:5:50) was added at 37 °C for 1 h in the dark. Slides were washed with PBS and stained with antifluorescence-quenching sealing liquid containing DAPI before images were acquired with an inverted fluorescence microscope (Leica Biosystems, Wetzlar, Germany).

### 2.6. Immunohistochemistry

Paraffin-embedded samples of 3–5 μm were dewaxed and rehydrated before the addition of ethylenediaminetetraacetic acid (EDTA) antigen retrieval solution and microwave antigen retrieval of aldehyde-fixed paraffin-embedded sections. H_2_O_2_ and 4% bovine serum albumin were used to block the endogenous peroxidase and nonspecific antigens and sections were incubated with rabbit anti-CD80 and mouse anti-CD86 antibodies (1:500 dilution) at 4 °C overnight, followed by washing and incubation with HRP-conjugated anti-IgG (1:500 dilution) at room temperature for 30 min. Sections were stained with diaminobenzidine (DAB) and counterstained with hematoxylin solution for dehydration and mounting.

### 2.7. Immunofluorescence

DC2.4 cells were seeded into 12-well plates at a density of 1 × 10^5^ cells/mL and pretreated with 10 nM vitamin D_3_ for 12 h followed by treatment with 10 μg/mL LPS and Brefeldin A for 24 h. Cells were washed with 1X PBS buffer and fixed with 4% paraformaldehyde for 15 min at room temperature. The cells were permeabilized with 0.5% Triton X-100 for 20 min and then blocked with 5% BSA for 30 min at room temperature. Antibodies raised against CD80, CD86, IDO-1, TNF-α, IL-1β, and iNOS were added to cells for incubation at 4 °C overnight, after which cells were washed and incubated with Cy3-conjugated IgG secondary antibodies for 1 h in the dark. Cells were washed and stained with antifluorescence-quenching sealing liquid containing DAPI for 5 min for observation under an inverted fluorescence microscope. Mean fluorescence intensity was quantified using ImageJ software.

### 2.8. Quantitative real-time PCR assay

DC2.4 cells were seeded into 12-well plates at a density of 1 × 10^5^ cells/mL and pretreated with 10 nM vitamin D_3_ for 12 h followed by 10 μg/mL LPS for 24 h. Total RNA was extracted using the TRIeasy Total RNA Extraction Reagent and cDNA was synthesized using Hifair III 1st Strand cDNA Synthesis SuperMix. Real-time PCR was performed using Hieff qPCR SYBR Green Master Mix and gene-specific primers designed by Primer 3 software (Table 1) on a TL988 Real-Time PCR system (TIANLONG, Xi’an, China). Relative gene expression was calculated using the −2^ΔΔCt^ method with β-actin as the internal reference.

### 2.9. FITC-OVA uptake assay

DC2.4 cells were seeded into 12-well plates at a density of 1 × 10^5^ cells/mL and pretreated with 10 nM vitamin D_3_ for 12 h followed by treatment with 10 μg/mL LPS for 24 h and incubated with 2 μg/mL ovalbumin (OVA)-FITC at 37 °C for 30 min. Cells were washed and endocytosis was observed and photographed under an inverted fluorescence microscope.

### 2.10. Statistical analysis

Data were expressed as mean ± standard deviation (SD) and GraphPad Prism 9 software was used for statistical analysis. For normally distributed continuous variables, we summarized the data as mean and SD values, and comparisons between groups were performed using Student’s t-test and ANOVA. For continuous data exhibiting skewed distribution, differences in the data were tested using the Mann–Whitney U test or Kruskal–Wallis test. Categorical data were summarized as counts with relative frequencies as percentages. Differences in the groups were analyzed using the chi-square test or Fisher’s exact test (when the count was less than 5). Values of p < 0.05 were considered statistically significant.

## 3. Results

### 3.1. Effect of vitamin D_3_ on intestinal damage in a neonatal rat model of NEC

**Figure 1:**
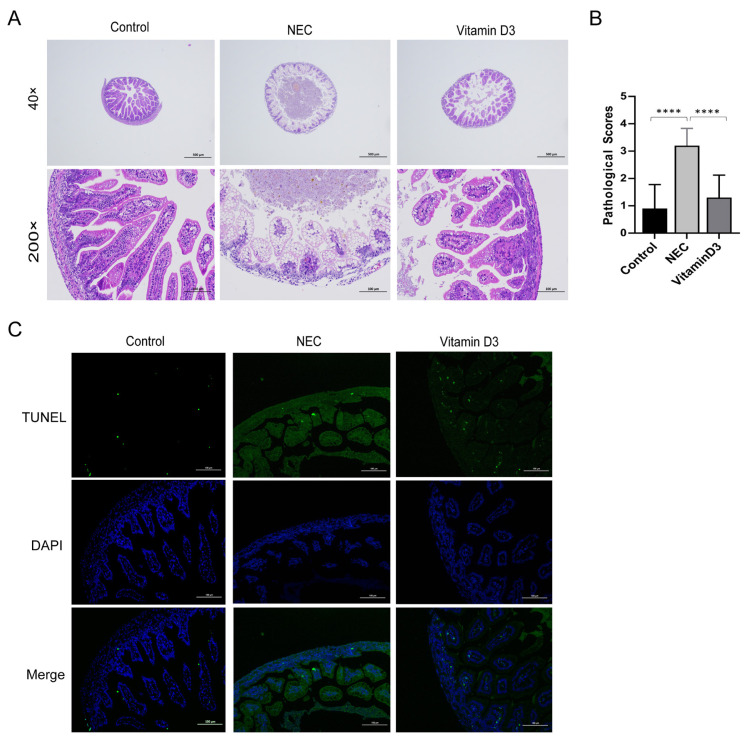
Effect of vitamin D_3_ on intestinal damage in a neonatal rat model of NEC.

Oral vitamin D_3_ was given to rats with NEC, and intestinal specimens were taken for H&E staining and graded according to Barlow’s protocol. Samples with an intact intestinal structure with a complete and continuous epithelium, a neat arrangement of glands, and lamina propria and submucosa that were free of hyperemia and edema were seen in control specimens and given a pathological score of 0. In contrast, severe intestinal necrosis with villus degeneration and edema, partial villus necrosis, disordered glands, edema of the lamina propria and submucosa, and thin and broken myometrium were seen in samples from the rats with NEC, which were given pathological scores of 3 or 4. However, neonatal NEC rats that had been treated with vitamin D_3_ had only slight edema of the lamina propria and submucosa, with irregular villi and glands with slight intestinal lesions, and these samples received pathological scores of 0 or 1 (Table 2; Figure 1A). Thus, the pathological scores of the NEC group were higher than those of the controls and the vitamin D_3_-treated animals. The vitamin D_3_-treated group and the NEC group both received higher pathological scores than the control group (p < 0.0001; Figure 1B). TUNEL measurements showed that apoptosis of tissues in the NEC group was more significant than that in the control group and the samples from rats treated with vitamin D_3_ (Figure 1C).

### 3.2. Effect of vitamin D_3_ on the expression of immune cell costimulatory molecules

**Figure 2:**
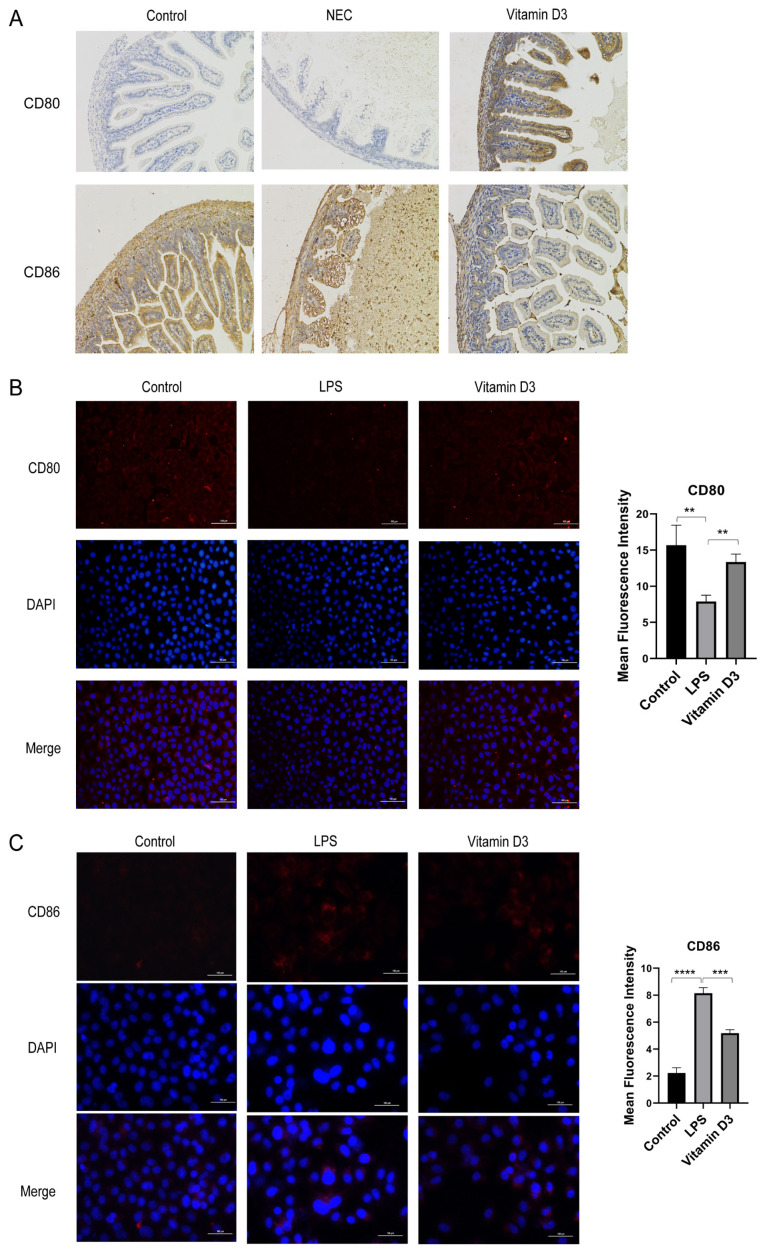
Effect of vitamin D_3_ on expression of costimulatory molecules in immune cells.

Levels of CD80 (B7-1) and CD86 (B7-2) in DCs were analyzed by immunohistochemical techniques. CD80 was expressed at basal levels in control and NEC model intestinal tissues but was increased in the tissues obtained from vitamin D_3_-treated animals. The reverse was true for CD86, which was highly expressed in the control and NEC groups but low in vitamin D_3_-treated rats (Figure 2A). To provide further evidence of the effect of vitamin D_3_ on the function of DCs, mouse dendritic DC2.4 cells were pretreated with vitamin D_3_ before exposure to LPS, and CD80 was found to be downregulated by LPS treatment and restored by vitamin D_3_ (p < 0.01; Figure 2B). The expression of CD86 was increased after LPS treatment and alleviated by vitamin D_3_ (p < 0.001; Figure 2C).

### 3.3. mRNA expression

**Figure 3:**
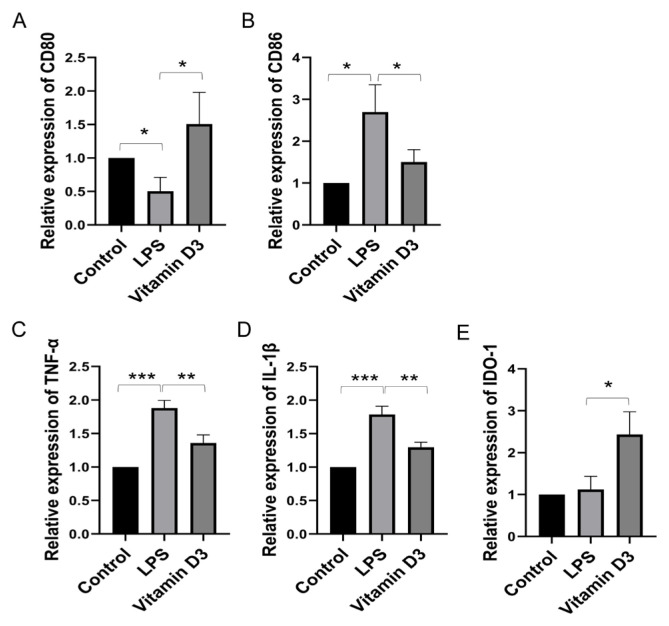
Effect of vitamin D_3_ on the expression of inflammatory factors, costimulatory molecules, and immunoregulatory genes.

Expressions of mRNA corresponding to TNF-α, IL-1β, IDO-1, CD80, and CD86 were measured by qPCR. CD80 expression decreased after LPS treatment and recovered with vitamin D_3_, while the opposite pattern was seen for CD86 (p < 0.05; Figures 3A and 3B). Expression levels of inflammatory factors TNF-α and IL-1β were increased in DC2.4 cells after LPS treatment (p < 0.001) and this effect was reversed by vitamin D_3_ treatment (p < 0.01). LPS also induced the expression of IDO-1, an indicator of the tolerogenic DC phenotype, and this factor was further upregulated by vitamin D_3_ (p < 0.05; Figures 3C–3E).

### 3.4. Effect of vitamin D_3_ on LPS-induced maturation and activation of DC2.4 cells

**Figure 4:**
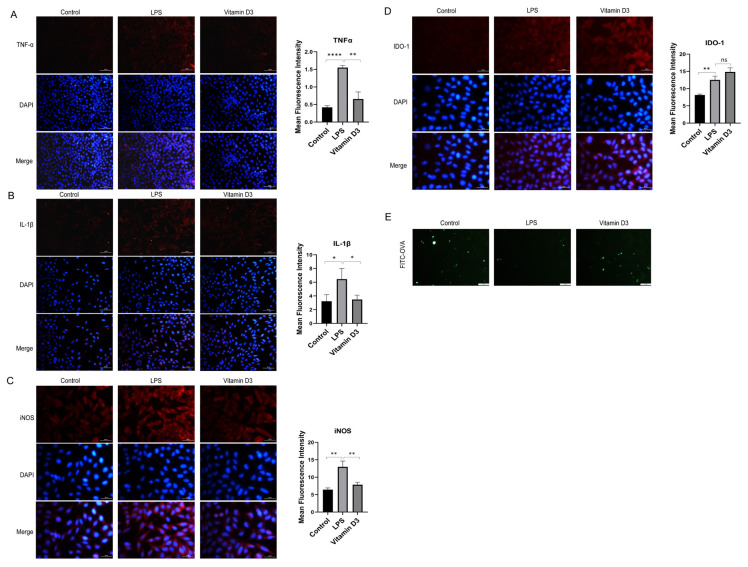
Effect of vitamin D_3_ on the maturation of LPS-treated DC2.4 cells and expression of cytokines.

Immunofluorescent measurements showed that the expression levels of TNF-α, IL-1β, and iNOS in DC2.4 cells increased after LPS treatment (p < 0.0001, p < 0.05, and p < 0.01) and these levels were reduced with vitamin D_3_ (p < 0.01, p < 0.05, and p < 0.01) (Figures 4A–4C). Thus, vitamin D_3_ appears to inhibit the LPS-induced inflammatory response in DC cells. DC cells induce tolerogenic antigen-presenting cells in addition to controlling infection and promoting inflammation. IDO-1 mediates maternal tolerance of fetal alloantigens and influences the differentiation of regulatory T cell (Tregs). IDO-1 expression increased with LPS (p < 0.01), an effect that was especially pronounced in the vitamin D_3_-treated group (Figure 4D). FITC-OVA uptake assays showed a reduced capacity of soluble antigen uptake by DC2.4 cells after LPS treatment, a reduction that showed partial recovery with vitamin D_3_. Thus, vitamin D_3_ appears to preserve the immature state of DCs (Figure 4E).

## 4. Discussion

Excessive inflammatory response to luminal microbial stimuli has been observed in NEC animal models and clinical cases and may aggravate intestinal barrier damage [28]. Increased TLR4 expression has been reported in NEC animal models and resected intestines from infants, and it is acknowledged that nondigestible oligosaccharides present in breast milk may prevent NEC by inhibition of TLR4 through the Wnt/Notch pathway, contributing to epithelial maturation [29,30]. The release of inflammatory mediators by the damaged intestinal epithelium leads to the infiltration of leukocytes, including neutrophils, macrophages, and DCs. The interaction of pathogen-associated molecular patterns with surface receptors of DCs embedded in the epithelium causes DC maturation and DC-mediated differentiation of naive T cells [31]. Probiotics are considered to promote the generation of tolerogenic DCs, which influence Treg proliferation and differentiation and promote immune homeostasis [32,33]. Our study found that the effect of vitamin D_3_ on rats with NEC is exerted through alleviation of intestinal damage, manifested by improved intestinal integrity and reduced numbers of apoptotic cells. Vitamin D_3_ also altered the intestinal expression of CD80/CD86, in line with treatment of the DC2.4 cells with LPS and vitamin D_3_. CD80/CD86 are B7 family ligands, largely restricted to DCs and B cells, and they share two receptors, CD28 and CTLA-4, which show competitive ligand binding and regulate T-cell activation and tolerance [34]. CD80 binds both receptors with a higher affinity than CD86, but CTLA-4, expressed on Treg cells, depletes CD80/CD86 by transendocytosis and induces IDO expression in antigen-presenting cells, leading to reduced CD28-mediated T cell activation [35,36].

The individual roles of CD80 and CD86 remain poorly characterized. The two proteins showed different expression changes in intestinal tissues, and altered DC2.4 cell phenotypes were consistent with those in intestinal tissue. Vitamin D_3_ inhibited the expression of LPS-induced inflammatory factors and increased that of IDO-1 in DC2.4 cells. A decrease in CD86 expression is thought to suppress DC maturation, prolonging allograft survival in heart-transplanted mice and relieving RSV-induced asthma [37,38]. IDO is a tryptophan-catabolizing enzyme that is predominantly expressed in innate immune cells, such as macrophages and DCs, where it catabolizes and depletes the tryptophan required for T-cell proliferation [39]. IDO gene transcription is regulated by inflammatory mediators and interferon (IFN)-sensitive elements are present in the promoter regions of both mouse and human IDO genes [40]. LPS and inflammatory cytokines enhance IDO expression synergistically with IFN-γ in vitro and soluble CTLA4-immunoglobulin binds CD80/CD86 to induce IDO expression [41,42]. IDO is considered to regulate DC maturation and antigen-presenting cell-mediated T-cell suppression. IDO inhibited CD86 expression and upregulated DC PD-L1, and IDO-deficient DCs promoted the differentiation of T cells towards a proinflammatory phenotype in a previous study [43]. LPS increased IDO-1 expression and decreased endocytosis in the DC2.4 cells of the current study and vitamin D_3_ further upregulated IDO-1 and restored the endocytic capacity. These in vitro results indicate that vitamin D_3_ regulated IDO-1 and CD80/CD86 expression to influence DC maturation.

There are some limitations to the current study. The mechanism by which vitamin D_3_ regulates CD80/CD86 and IDO-1 expression remains unclear and it has not been established whether the observed attenuation of intestinal damage in NEC rats was dependent on altered CD80/CD86 and IDO-1 expression. Further study of vitamin D_3_ function in intestinal epithelial cell lines and animal models in vivo is required.

In conclusion, vitamin D_3_ has been demonstrated to influence DC maturation and activation, probably through an effect on CD80/CD86 and IDO-1 expression. Vitamin D_3_ decreased CD86 expression and increased that of CD80 and IDO-1, maintaining immature and inactive DC status. Further study of the mechanisms underlying vitamin D_3_ regulation of immune cell maturation, differentiation, and responses may have significance for disease prevention and adjuvant treatment.

## Figures and Tables

**Table 1:** Primers for qRT-PCR.

Primer	Sequence
Actin forward	GGTACCACCATGTACCCAGG
Actin reverse	AGGGTGTAAAACGCAGCTC
CD80 forward	CGTCGTCATCGTTGTCATCATC
CD80 reverse	AAGGAAGACGGTCTGTTCAGC
CD86 forward	TCCTCCTTGTGATGCTGCTC
CD86 reverse	CTGCATTTGGTTTTGCTGAAGC
TNF-α forward	GACACCATGAGCACAGAAAGC
TNF-α reverse	TTGGTGGTTTGCTACGACGT
IL-1β forward	GCACTACAGGCTCCGAGATGA
IL-1β reverse	TTGTTGGTTGATATTCTGTCCATTG
IDO-1 forward	CATCACCATGGCGTATGTGTG
IDO-1 forward	CCATTGGGGTCCTTTTTCTTCC

**Table 2:** Intestinal damage and pathological scores of the control groups, NEC groups, and vitamin D groups.

Groups	Intestinal damage	Pathological score
Control groups	Intact intestinal structure with complete and continuous epithelium, neat arrangement of glands, and lamina propria and submucosa free of hyperemia and edema	0
NEC groups	Severe intestinal necrosis with villus degeneration and edema, partial villus necrosis, disordered glands, edema of the lamina propria and submucosa, and thin and broken myometrium	3 or 4
Vitamin D_3_ groups	Only slight edema of the lamina propria and submucosa, irregular villi and glands with slight intestinal lesions	0 or 1

## Data Availability

The corresponding author can provide the data used to support this study upon request.
